# Left Atrial Diverticula Detected on Cardiac CT in Patients With Acute Ischemic Stroke: A Substudy of Mind the Heart

**DOI:** 10.1161/STROKEAHA.125.051199

**Published:** 2025-07-07

**Authors:** Shan Sui Nio, Leon A. Rinkel, Olivia N. Cramer, Z. Beyda Özata, Chiel F.P. Beemsterboer, Valeria Guglielmi, Berto J. Bouma, S. Matthijs Boekholdt, Nick H.J. Lobé, Ludo F.M. Beenen, Nerea Arrarte Terreros, Henk A. Marquering, Charles B.L.M. Majoie, Yvo B.W.E.M. Roos, Adrienne van Randen, R. Nils Planken, Jonathan M. Coutinho

**Affiliations:** Departments of Neurology (S.S.N., L.A.R., O.N.C., Z.B.O., C.F.P.B., V.G., Y.B.W.E.M.R., J.M.C.) Amsterdam UMC Location University of Amsterdam, the Netherlands.; Departments of Cardiology (B.J.B., S.M.B.) Amsterdam UMC Location University of Amsterdam, the Netherlands.; Departments of Radiology and Nuclear Medicine (N.H.J.L., L.F.M.B., N.A.T., H.A.M., C.B.L.M.M., A.v.R.) Amsterdam UMC Location University of Amsterdam, the Netherlands.; Departments of Biomedical Engineering and Physics (N.A.T., H.A.M.) Amsterdam UMC Location University of Amsterdam, the Netherlands.; Departments of Digital Health and Biomedical Technologies, Vicomtech, Spain (N.A.T.).; Departments of Radiology, Mayo Clinic, Rochester, MN (R.N.P.).

**Keywords:** anticoagulants, diverticulum, heart atria, ischemic stroke, risk factors, tomography

## Abstract

**BACKGROUND:**

Left atrial (LA) diverticula are focal outpouchings of the LA wall and may increase ischemic stroke risk. We studied recurrent ischemic stroke in patients with acute ischemic stroke and LA diverticula detected on cardiac computed tomography.

**METHODS:**

We used data from a prospective cohort study of patients with acute ischemic stroke undergoing cardiac computed tomography acquired during the acute stroke imaging protocol. Cardiac radiologists systematically assessed the presence and characteristics of LA diverticula, defined as LA wall outpouchings with a length/ostium width ratio >0.5 and length ≥3 mm. We compared recurrent ischemic stroke and functional outcome (modified Rankin Scale) after 2 years between patients with and without LA diverticula, adjusting for age, history of atrial fibrillation and ischemic stroke, and anticoagulation use.

**RESULTS:**

Of 447 included patients (median age 72 [interquartile range (IQR), 62–81], 59% male), 126 (28%) had LA diverticula: median length 6 mm (IQR, 4–8), width 5 mm (IQR, 4–7), and volume 113 mm^3^ (IQR, 52–254). There was no statistically significant difference in age (median 70 [IQR, 58–79] versus 73 [IQR, 63–81]; *P*=0.06), sex (64% versus 57% male; *P*=0.28), history of ischemic stroke (21% versus 17%; *P*=0.48), atrial fibrillation (11% versus 19%; *P*=0.09), or baseline National Institutes of Health Stroke Scale score (median 5 [IQR, 2–14] versus 5 [IQR, 3–14]; *P*=0.54) between patients with and without LA diverticula, respectively. Recurrent ischemic stroke was more common in patients with LA diverticula (18/124 [15%] versus 24/314 [8%], adjusted hazard ratio, 2.01 [95% CI, 1.08–3.77]), and recurrence risk increased with diverticulum volume (adjusted hazard ratio, 1.02 [95% CI, 1.01–1.03] per 10 mm^3^). Functional outcome was better in patients with diverticula (median modified Rankin Scale score of 2 [IQR, 1–3] versus 3 [IQR, 1–6], adjusted common odds ratio, 0.62, [95% CI, 0.42–0.92]).

**CONCLUSIONS:**

LA diverticula are a common finding on cardiac computed tomography in patients with acute ischemic stroke, and the risk of recurrent ischemic stroke was increased in these patients, particularly in those with larger diverticula. Atrial diverticula may be a risk factor for recurrent ischemic stroke.

**REGISTRATION:**

URL: https://www.onderzoekmetmensen.nl/nl/trial/50352; Unique identifier: NL6413901818.

Left atrial (LA) diverticula are focal outpouchings of the myocardium of the left atrium.^1^ LA diverticula typically seem as sac-like, smooth-walled pouches with a wide neck that communicate with the left atrium and can vary in shape and size.^2,3^ The cause of LA diverticula is not fully understood, and their origin may be either congenital or acquired. LA diverticula may develop when a section of the muscular atrial wall weakens, leading to outward movement of these weakened areas due to pressure on the wall.^3–6^ Congenital LA diverticula may be a result from the embryological common pulmonary vein^2,7^ or incomplete regression of cardinal veins.^8^ They develop from congenitally weak areas of the atrial wall and dilate due to increasing atrial blood flow and pressure.^9,10^ At an older age, LA diverticula may be acquired due to pathology such as myocardial infarction, systemic thrombosis or arrhythmia.^3,5^ Wan et al^3^ described that these conditions can lead to weakening and thinning of the atrial wall and subsequent protrusion, as the arteries that supply the atrial muscle may become occluded. The prevalence of LA diverticula detected by cardiac computed tomography (CT) varies between 17% and 38% in patients undergoing cardiac CT for diverse reasons,^1^ and they are often reported as an incidental finding. Echocardiography is the most widely used diagnostic modality to detect cardioembolic sources of stroke.^11^ However, echocardiography, and especially transthoracic echocardiography, may not always detect small abnormalities such as LA diverticula,^9^ and only a few case reports have described the presence of diverticula using transthoracic^6,12–14^ and transesophageal echocardiography.^15,16^ Cardiac CT provides detailed images of cardiac anatomic structures with high temporal and spatial resolution. As cardiac CT is increasingly used to detect cardioembolic sources of stroke, LA diverticula will likely be more frequently detected in stroke patients.^17–20^

The clinical relevance of LA diverticula is unclear. It has been hypothesized that LA diverticula may act as an ectopic focus for arrhythmias, increasing the risk of atrial fibrillation (AF) and therefore thromboembolic events.^21,22^ It has also been suggested that the outpouching of LA diverticula could cause low-velocity recirculation, promoting blood stasis that could potentially lead to thrombus formation, which has also been confirmed in case reports.^6,23^ On the other hand, other studies did not find an association between LA diverticula and ischemic brain lesions on magnetic resonance imaging^24,25^ or history of ischemic stroke.^1,26^ A recent meta-analysis of 15 studies investigated the prevalence of LA diverticula and accessory left atrial appendages (LAAs), detected on CT imaging. This study reported a thrombus within 0.2% of these additional LA structures in 984 patients. However, they did not separately report the number of thrombi detected in LA diverticula.^5^

There is a lack of data on LA diverticula in patients with ischemic stroke. Therefore, in the current study, we examined the prevalence, morphology, size, location, and clinical relevance of LA diverticula detected on cardiac CT in patients with acute ischemic stroke (AIS).

## Methods

The data will be made available upon reasonable request to the corresponding author.

This was a post hoc analysis of the Mind the Heart study, a single-center, prospective, observational, cohort study, conducted at the Amsterdam University Medical Center (location Academic Medical Center) between 2018 and 2020. The Mind the Heart study included adult patients with AIS who underwent cardiac CT acquired during the acute stroke imaging protocol.^19,27^ Patients with a transient ischemic attack or a stroke mimic were excluded. Patients underwent a noncontrast enhanced brain CT, nongated CT angiography of the brain, cervical arteries, and aortic arch, and perfusion CT when applicable, followed by a prospective ECG-gated cardiac CT. As part of routine clinical care, ECG, rhythm monitoring (for 48–72 hours), and transthoracic echocardiography were performed either during hospital admission or outpatient follow-up. Stroke etiology was determined based on the TOAST (Trial of ORG 10172 in Acute Stroke Treatment) criteria.^28^ We included the results of the cardiac CT to determine stroke etiology. LA diverticula were not considered as a cardioembolic etiology for the TOAST classification.

**Figure 1.**
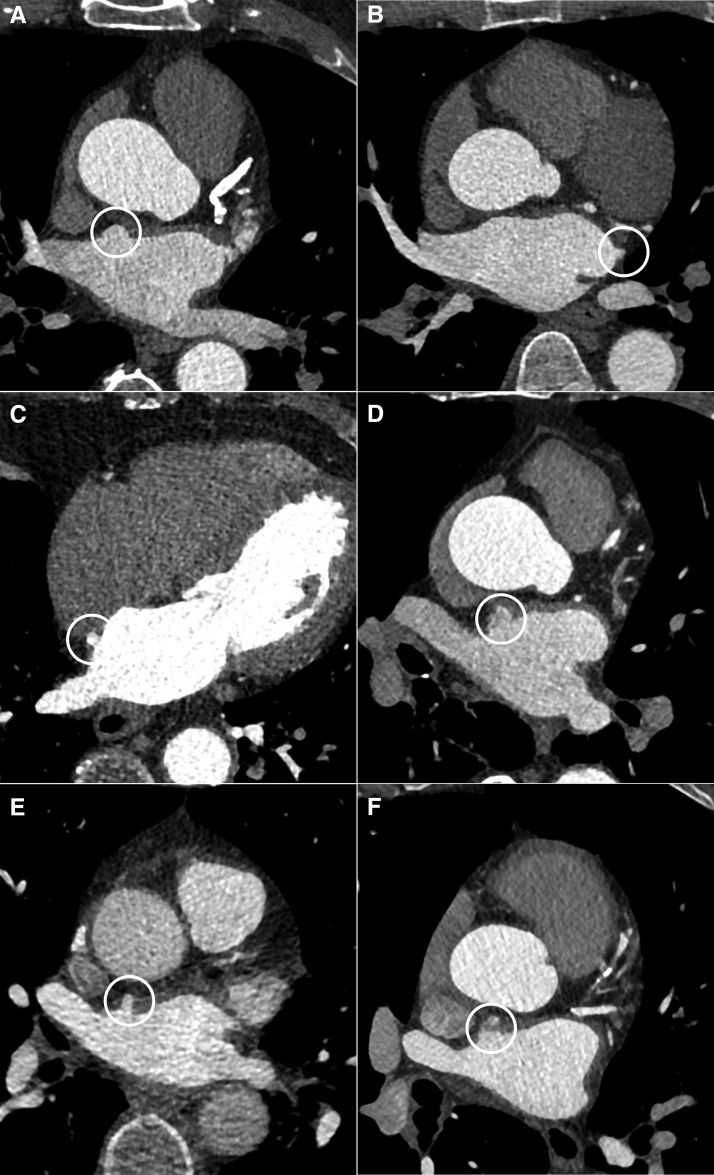
**Axial images of acute cardiac computed tomography portraying left atrial diverticula. A**, Right superior location with a cystic shape, (**B**) left superior location with a cystic shape, (**C**) right inferior location with cystic shape, (**D**) trabeculated endocardial surface (**E**) tubular shape (**F**) right superior location with hypodensity within outpouching, suggesting slow-flow (Hounsfield units >100).

The presence of LA diverticula on cardiac CT was evaluated by an experienced cardiac radiologists (R.N.P. and A.v.R.) using axial, sagittal, coronal, and multiplanar reconstructions. We defined LA diverticula as an outpouch of the LA wall^1^ and included only those with a ratio (diverticulum length/ostium width) >0.5 and a minimum diverticulum length of ≥3 mm to enhance classification and exclude minor variations in cardiac contour. Each diverticulum was assessed for length, width, ostium width, location, shape, endocardial surface, and the presence of a thrombus within the diverticulum. Figure 1 shows several examples of LA diverticula on cardiac CT. Diverticulum length was measured along its long axis at the point where the diverticulum diameter was widest (distance from ostium to apex).^29,30^ Diverticulum width was measured perpendicular to the long axis at the widest diameter (parallel to the ostium at the widest diameter).^4,29,30^ Ostium width was defined as the diameter of the diverticulum ostium opening. We calculated diverticulum volume using the formula for a cylinder: π×(diverticulum width/2)^2^×diverticulum length. To assess the diverticulum location, we divided the atrium into 4 subsections: superior and inferior, right and left. We classified diverticulum shape in 2 different categories: cystiform (diverticulum length/ostium width is ≤2) and tubular (diverticulum length/ostium width is >2).^3,31^ In addition, we assessed the endocardial surface as smooth or irregular (if trabeculae were present).^10,31^ Patients in which image quality was insufficient, resulting in an inability to evaluate the presence of LA diverticula were excluded. In cases where the presence of a diverticulum was unclear, a second cardiac radiologist reviewed the images.

Follow-up was performed after 2 years using a structured interview. Trained medical staff performed the interview with the patient or their legal representative to assess functional status and the occurrence of new cardiovascular events during follow-up. We obtained additional information and confirmation regarding recurrent stroke events by contacting general practitioners or other treating physicians. Outcome events of interest were recurrent ischemic stroke events and functional outcome, measured using the modified Rankin Scale after 2 years.

### Statistical Analysis

We compared baseline characteristics, recurrent stroke, and functional outcome between AIS patients with LA diverticula and without LA diverticula. We compared continuous variables with a nonparametric Mann-Whitney *U* test, and categorical variables with a χ^2^ test or a Fisher exact test as appropriate. For patients with a history of AF, we calculated CHA_2_DS_2_VASc-scores,^32^ and compared these between patients with and without LA diverticula using a nonparametric Mann-Whitney *U* test. We used a Cox regression model to investigate the association between LA diverticula and recurrent ischemic stroke. We corrected for the following potential confounders: age, history of AF, history of ischemic stroke, and anticoagulation use at baseline. We used an ordinal logistic regression model to evaluate functional outcome after 2 years using the modified Rankin Scale. We corrected for the following confounders: age, history of AF, prestroke modified Rankin Scale score and anticoagulation use at baseline. We investigated the association between diverticulum volume and recurrent stroke using a binomial logistic regression model for each parameter and adjusted for the same confounders as in the primary analyses. In addition, we performed a post hoc analysis replacing the variable anticoagulation use at baseline with anticoagulation use at discharge in the regression model to assess whether it could act as a potential confounder in the association between recurrent ischemic stroke and LA diverticula. In addition, we performed a post hoc interaction analysis to investigate whether anticoagulation use at baseline modifies the effect of LA diverticula on the outcome measures by incorporating an interaction term into the regression models and by performing 2 subgroup analyses. The first subgroup analysis separated patients with and without LA diverticula and stratified them by anticoagulation use to investigate a different effect of LA diverticula on the outcome measures between patients with and without anticoagulation use. The second analysis included the total population stratified by anticoagulation use to investigate the outcome measures between patients with and without anticoagulation use. We adjusted for the same potential confounders as the main analysis, excluding anticoagulation use. Analyses were performed using R, version 4.4.2 (R foundation for Statistical Computing 2023). For all tests, we used a 2-sided significance level of 0.05.

### Standard Protocol Approvals, Registrations, and Patient Consents

All patients or their legal representatives provided written informed consent, and the study was approved by the medical ethics committee of Amsterdam UMC, location Academic Medical Center (2018_017C2018275). The procedures followed were in accordance with institutional guidelines. S.S.N. had full access to all the study data and takes responsibility for the integrity of the study and the data analysis. This study was reported according to the Strengthening the Reporting of Observational Studies in Epidemiology guidelines.^33^ The study protocol was registered on December 24, 2020, with the Dutch Central Committee on Research Involving Human Subjects (CCMO; NL6413901818, https://www.onderzoekmetmensen.nl/nl/trial/50352). The protocol and main results have been published.^19,27^

## Results

**Figure 2.**
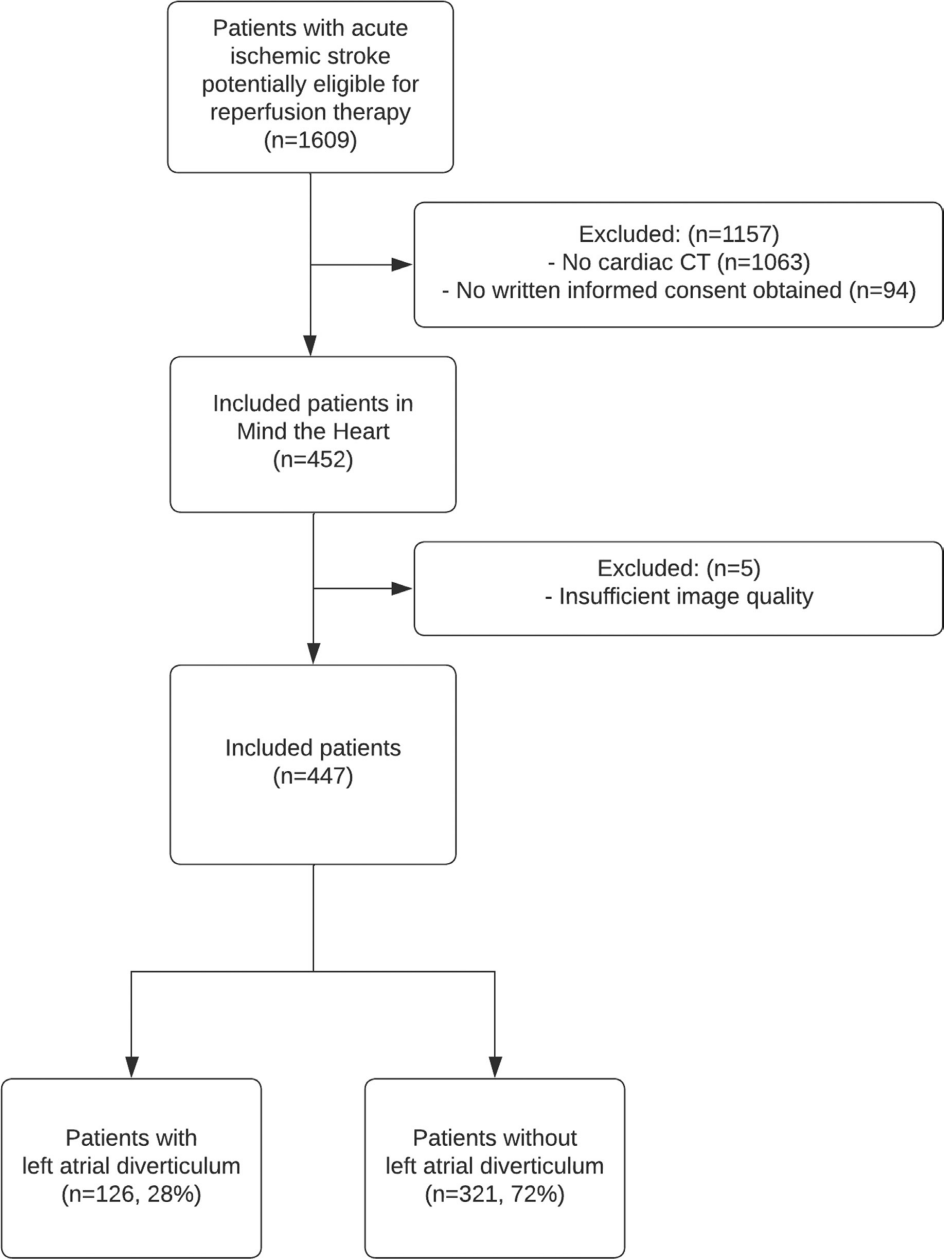
**Flowchart of included patients.** CT indicates computed tomography.

A total of 452 patients with AIS were included in the Mind the Heart study. Five patients were excluded because the image quality of the cardiac CT was insufficient for detecting LA diverticula, leaving 447 patients for assessment: median age 72 (interquartile range [IQR], 62–81), 264 (59%) male. Of these, 126 (28%) patients had an LA diverticulum (Figure 2). Of 131 cases initially classified as LA diverticula, 19 cases were considered uncertain and required a secondary reading by a radiologist. This resulted in 5 cases being reclassified as no LA diverticula based on the presented definition, resulting in a total of 126 patients with an LA diverticulum.

Baseline characteristics in patients with and without LA diverticula were largely comparable (Table 1). Specifically, there were no significant differences in age (median 70 [IQR, 58–79] versus 73 [IQR, 63–81]; *P*=0.06), sex (80 [64%] versus 184 [57%] male; *P*=0.28), National Institutes of Health Stroke Scale score at baseline (median 5 [IQR, 2–14] versus 5 [IQR, 3–14]; *P*=0.54), previous stroke (27 [21%] versus 55 [17%]; *P*=0.48), AF (14 [11%] versus 62 [19%]; *P*=0.09), or myocardial infarction (16 [13%] versus 42 [13%]; *P*=0.82) between patients with and without LA diverticula, respectively. Patients with LA diverticula less often used anticoagulation at baseline (12 [10%] versus 67 [21%]; *P*=0.007). A thrombus in the LAA, LA, or left ventricle was reported in 10 patients (8%) with LA diverticula (8 LAA, 1 left ventricular, and 1 patient with a thrombus in both the LAA and LA) and 28 patients (9%) without LA diverticula (21 LAA, 6 left ventricular, and 1 patient with a thrombus in both the LAA and LA). The median CHA_2_-DS_2_-VASc scores for patients with AF and LA diverticula compared with patients without diverticula were 4 (IQR, 2–4) and 4 (IQR, 3–4); *P*=0.84, respectively. Given the limited number of patients with AF, especially in patients with LA diverticula (n=14), we have refrained from including the CHA_2_-DS_2_-VASc scores in the regression models.

**Table 1.**
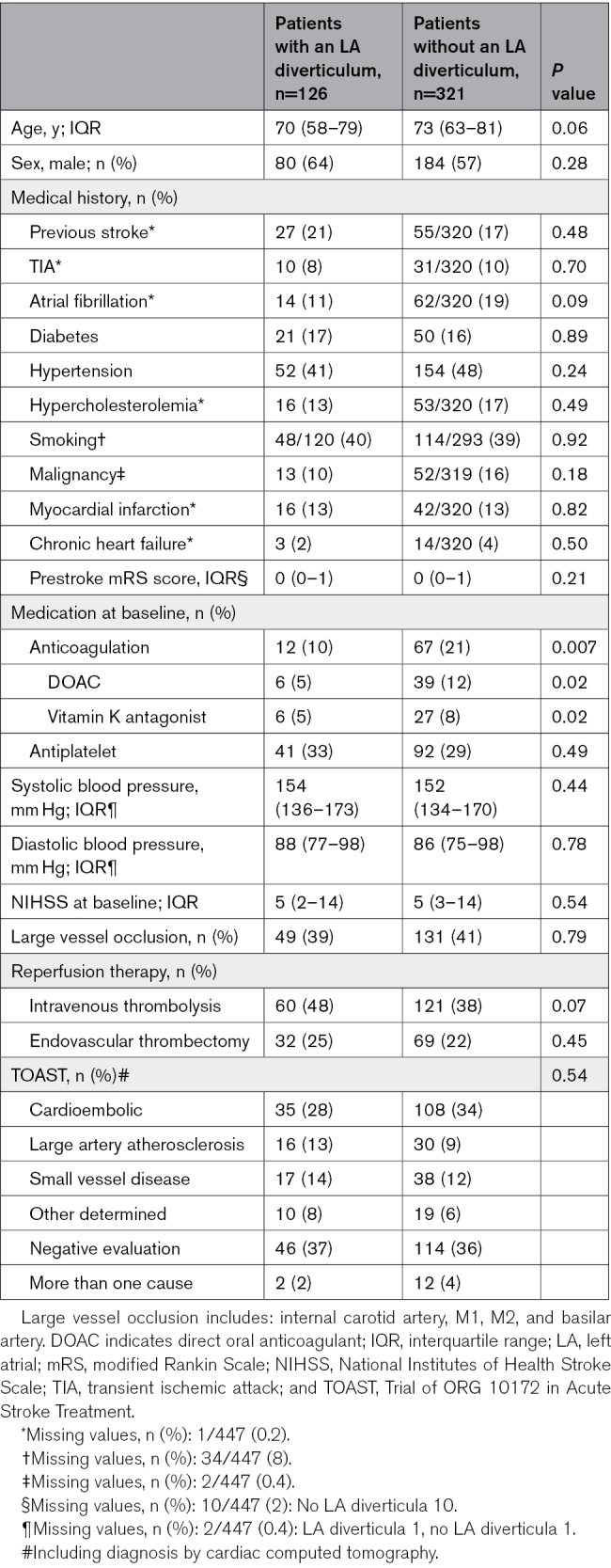
Baseline Characteristics

Table 2 shows an overview of LA diverticulum characteristics. Of the patients with an LA diverticulum, 112 (89%) had 1 diverticulum and 14 (11%) had 2 diverticula. Diverticulum characteristics were as follows: median length 6 (IQR, 4–8) mm, width 5 (IQR, 4–7) mm, ostium width 5 (IQR, 3–7) mm and volume 113 (IQR, 52–254) mm^3^. The most common location was right superior in 105 (83%) patients followed by right inferior in 14 (11%) patients, and 103 (82%) were classified as cystic and 23 (18%) as tubular. LA diverticula had a smooth endocardial surface in 104 (83%) cases and an irregular surface in 22 (17%). There was no thrombus detected within a diverticulum.

**Table 2.**
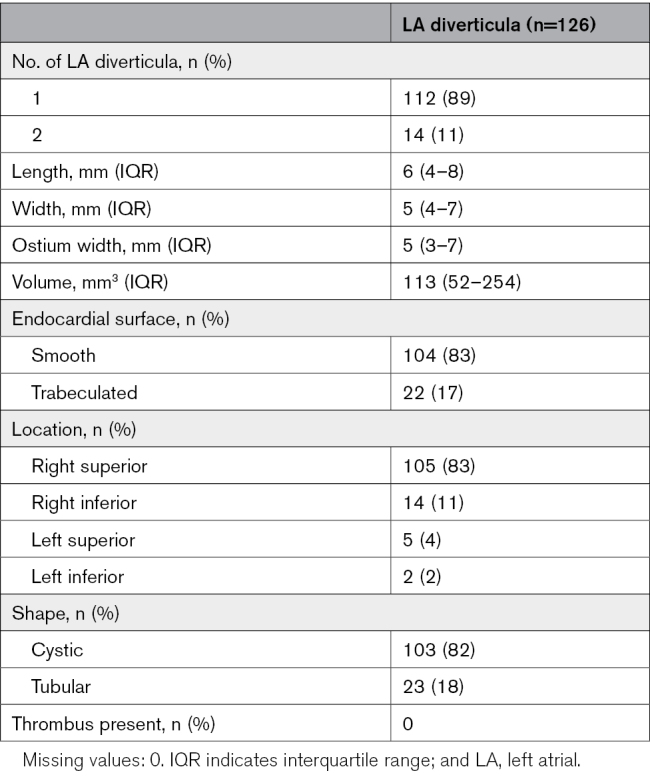
Overview Characteristics of LA Diverticula

**Figure 3.**
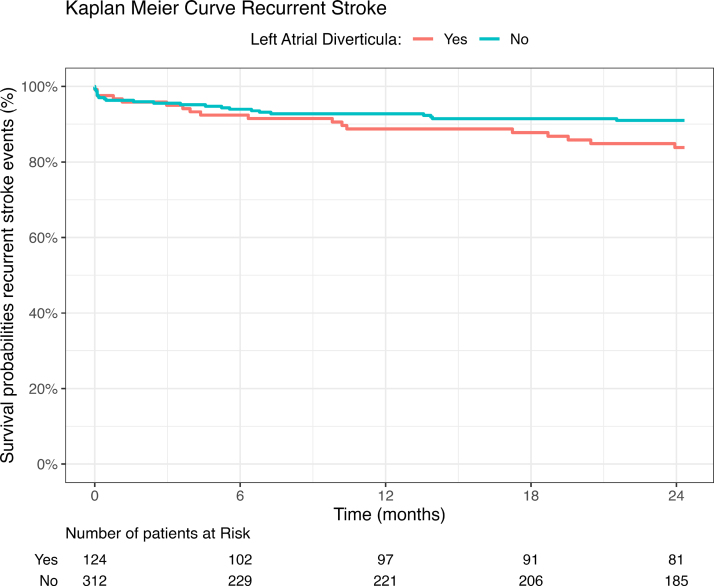
**Survival analysis between left atrial (LA) diverticula and recurrent stroke.** Cox regression analysis of LA diverticula vs no LA diverticula (adjusted hazard ratio, 2.01 [95% CI, 1.08–3.77]; *P*=0.03). We corrected for the following potential confounders: age, history of atrial fibrillation, history of ischemic stroke, and anticoagulation use at baseline.

Patients with LA diverticula had a higher risk of recurrent ischemic stroke (15% versus 8%, adjusted hazard ratio, 2.01 [95% CI, 1.08–3.77], *P*=0.03; Table 3 and Figure 3). Of the patients with an LA diverticulum and a recurrent stroke, 4 of 18 (22%) used anticoagulants, and 14 of 18 (78%) used antiplatelets at the time of recurrent stroke (Table 4). There was a significant association between recurrent stroke and increasing diverticulum volume (adjusted hazard ratio, 1.02 [95% CI, 1.01–1.03] per 10 mm^3^, *P*=0.001; Table 5). Table 4 shows the diverticulum characteristics stratified by recurrent stroke. No recurrent stroke occurred in patients with more than 1 diverticulum. The etiologies of the recurrent strokes included: 8 cardioembolic (44%), 8 unknown (44%), 1 small vessel disease (6%), and 1 large artery atherosclerosis (6%) in patients with LA diverticula; and 12 cardioembolic (50%), 4 large artery atherosclerosis (17%), 3 small vessel disease (13%), and 5 unknown (21%) in patients without LA diverticula.

**Table 3.**
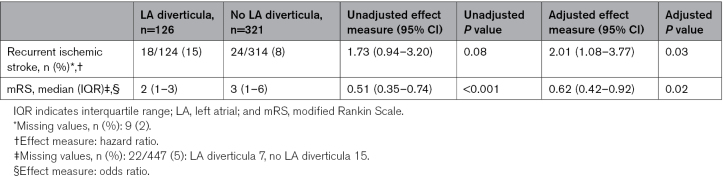
Outcomes at 2-Year Follow-Up

**Table 4.**
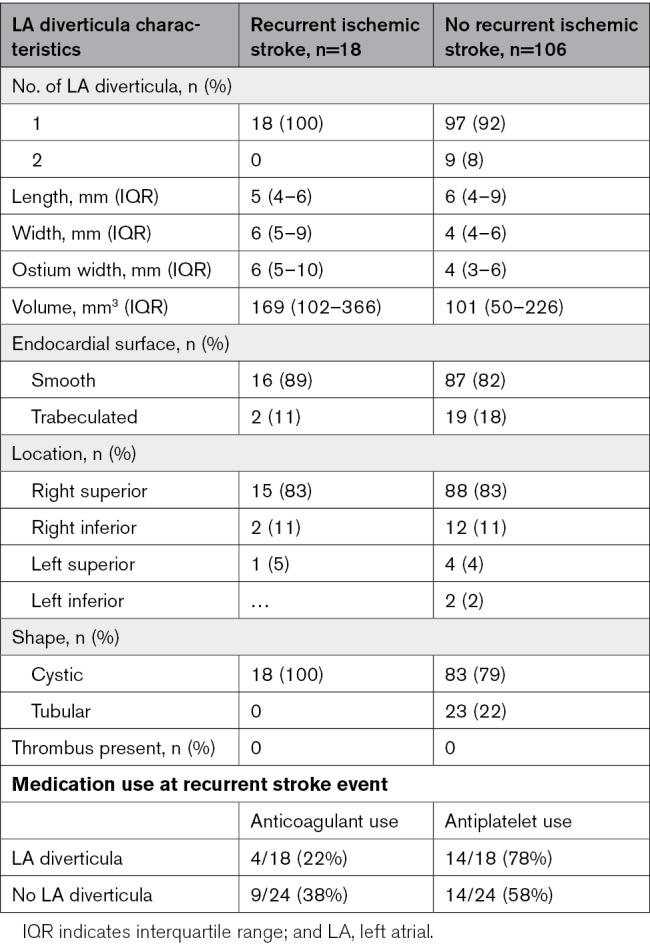
Overview Characteristics of LA Diverticula Stratified by Recurrent Ischemic Stroke and Medication Use at the Moment of Recurrent Stroke Event

**Table 5.**
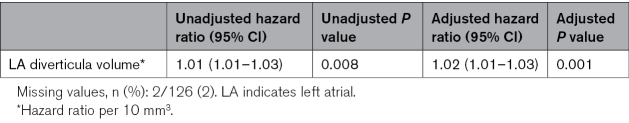
Association Between LA Diverticulum Volume and Recurrent Ischemic Stroke

The presence of an LA diverticulum was associated with better functional outcome after 2 years: median modified Rankin Scale score of 2 (IQR, 1–3) versus 3 (IQR, 1–6), adjusted common odds ratio, 0.62 (95% CI, 0.42–0.92), *P*=0.02 (Table 3 and Figure S1). There was no statistically significant difference in new AF diagnosed during admission or follow-up between patients with and without LA diverticula (25 [20%] versus 42 [13%]; *P*=0.10, respectively).

The results of the post hoc analysis on the association between recurrent ischemic stroke and LA diverticula, including anticoagulation use at discharge as a potential confounder, are shown in Table S1. The post hoc interaction analysis showed no significant effect of anticoagulation use at baseline on the association between LA diverticula and recurrent ischemic stroke (Table S2). Tables S3 and S4 show the results of the subgroup analyses.

## Discussion

In this single-center prospective cohort study among patients with AIS, LA diverticula were detected on acute cardiac CT in approximately a quarter of patients. Patients with LA diverticula had a 2 times higher risk of recurrent stroke within 2 years than patients without a diverticulum. Moreover, a larger diverticulum volume was associated with stroke recurrence. Surprisingly, patients with LA diverticula had better functional outcomes after 2 years compared with patients without LA diverticula.

There are several reasons why LA diverticula may be associated with thromboembolic events. First, LA diverticula may serve as an ectopic focus for arrhythmias.^4,5,8,22^ Histopathology has shown that the diverticulum wall consists of myocardium and fascia, enabling it to contract,^25,34^ generate electrical activity and potentially act as an ectopic focus.^21^ However, the prevalence of LA diverticula did not significantly differ between patients with AF and sinus rhythm, neither in our study nor in previous studies.^30,35–37^ In addition, there was no significant difference in new AF diagnosed during admission or follow-up, which makes this association less likely. Second, LA diverticula have been suggested as a location for thrombus formation due to low-velocity recirculation, promoting blood stasis within the diverticulum’s outpouching.^29,37,38^ However, in our study, no thrombi were detected in the diverticula. It could be that the thrombi are too small to be detected on cardiac CT, or have embolized to the brain, causing the index stroke. Both contractile properties^22^ and thrombi^5,6^ have been reported particularly in larger LA diverticula, which may explain the association between recurrent stroke and increasing diverticulum volume in our study. The prevalence of history of ischemic stroke did not significantly differ between patients with and without LA diverticula, while the recurrent stroke rates did. A possible explanation may be a change in diverticulum size over time and, as a result, potentially the risk of stroke. As LA diverticula may enlarge over time, the risk of thrombus formation may increase. This could explain the occurrence of both the index stroke and recurrent stroke in our patient population, despite a similar history of stroke across groups.

There is limited evidence regarding the clinical relevance of LA diverticula. A case-control study reported no difference in prevalence of LA outpouching structures (LA diverticula and accessory LAA) between ischemic stroke patients and control patients with no history of stroke or transient ischemic attack.^37^ Previous retrospective studies investigated LA diverticula in patients who underwent cardiac CT or CT coronary angiography for various reasons, such as coronary artery disease, and found no association with history of stroke, transient ischemic attack, or AF,^1,26^ which is similar to our findings, or between LA diverticula and ischemic brain lesions on magnetic resonance imaging.^24,25^ The association we found with recurrent stroke may be due to differences in the study population, as we focused on patients with AIS and the risk of stroke recurrence. This may have led to a selected population of patients with LA diverticula who are at a higher risk of stroke.

Interestingly, patients with LA diverticula had a better functional outcome at follow-up than patients without LA diverticula, though the underlying reason is unclear. Risk factors such as hypertension, hypercholesterolemia, active malignancy, and chronic heart failure were all slightly more common in patients without LA diverticula, indicating the possibility of residual confounding; however, these differences were not significant. The use of anticoagulants was significantly higher in patients without LA diverticula compared with those with LA diverticula, while the prevalence of known AF did not significantly differ. We adjusted for anticoagulation use and history of AF in our analysis, but this may not have accounted for all potential cardiovascular burdens. Additionally, we need to consider that this is a chance finding.

In previous studies, the prevalence of LA diverticula on cardiac and coronary CT varied between 17% and 38%, and our estimate is within the range of this spectrum.^1^ The variation in prevalence may be attributed to several reasons. First, these were studies investigating patients who underwent cardiac CT for various reasons, which led to different study populations, such as patients with AF or chest pain. Second, differentiating diverticula from other LA malformations, such as aneurysms and left-sided septal pouches, may be challenging.^3,5,7,34^ The specific features of each of these abnormalities can be subtle, are not clearly defined, and lesions can feature multiple characteristics of different abnormalities, which could increase the risk of misclassification.^29^

There is little evidence about possible treatment of LA diverticula. Surgical therapy by removing the diverticulum can be considered for cases involving a giant diverticulum or a persisting thrombus within the diverticulum.^6,13^ One could hypothesize that patients with LA diverticula may benefit from anticoagulant therapy to prevent recurrent stroke, as thrombus formation from LA diverticula or cardiac arrhythmias could potentially be the underlying cause of recurrent stroke events. However, this hypothesis requires further investigation.

Two radiologists performed the assessments of the LA diverticula. In case of diagnostic uncertainty, a secondary reading was performed. Given the fact that a second reading in our study was required in a minority of cases, and in even fewer cases led to a change in classification of LA diverticula being present, we believe that 1 radiologist will suffice in practice when assessing the presence of LA diverticula.

Our study had several limitations. First, there is currently no universally accepted definition of LA diverticula. We used a pragmatic and clear definition, which we hope will help future investigators who study this subject and enhance reproducibility of our results. Our classification of LA diverticula as outpouches with a specific ratio excludes superficial broad LA outpouches, which we consider to be minor variations in cardiac contour. We calculated LA volume using the formula for a cylinder. While this formula provides an estimation of the actual volume, it carries a risk of underestimating or overestimating the actual volume. A previous cohort study reported a mean diverticulum volume of 415±541 mm^3^ using voxel-wise segmentation to calculate blood volume within the diverticulum.^25^ Compared with our median volume of 113 (IQR, 52–254) mm^3^, it appears we may be underestimating the volume. However, since there are differences in study population, imaging techniques and methodologies, making a direct comparison is challenging. Future research is needed to identify methods that most accurately measure diverticulum volume. Second, we did not perform pathological examination of the LA diverticula or functional assessments, such as contractility, which may have been helpful for defining and differentiating other LA malformations, such as septal pouches or aneurysms. However, by using a clear definition for LA diverticula, we aimed to minimize misclassification. While our study did not investigate other LA structures, exploring these in future studies with stroke patients would be of interest. Third, our study used single-center data and had a small sample size and low number of outcome events, which limits the statistical power. Larger, multicenter prospective studies are required to confirm our findings, and results of the current study should be interpreted as hypothesis-generating only.

## Conclusions

LA diverticula were detected on acute cardiac CT in approximately a quarter of patients with AIS. Recurrent stroke occurred more frequently in patients with an LA diverticulum, especially those with larger-sized LA diverticula. While LA diverticula may be a novel risk factor for recurrent stroke, further research is necessary to establish definite conclusions due to the novelty of this topic in stroke.

## Article Information

### Sources of Funding

Dr Rinkel was supported by a personal Dekker Junior Clinical Scientist Grant from the Dutch Heart Foundation.

### Disclosures

The author(s) declared the following potential conflicts of interest with respect to the research, authorship, and publication of this article: Dr Bouma has received grants from Abbott Fund. Dr Majoie has received research grants from CVON/Dutch Heart Foundation, European Commission, Healthcare Evaluation the Netherlands, Stryker Corporation and Boehringer Ingelheim (paid to institution). Drs Majoie, Marquering, and Roos are shareholders of Nicolab, a company that focuses on the use of artificial intelligence for medical image analysis. Dr Coutinho reports grants from Medtronic, Siemens, AstraZeneca, and Bayer outside the submitted work (paid to institution). Dr Coutinho has received compensation from Portola Pharmaceuticals, LLC, for consultant services (paid to employer). Dr Coutinho is shareholder for Founder. Drs Coutinho and Marquering are shareholders of TrianecT. Dr Terreros and Marquering are cofounders and shareholders of inSteps. The other authors report no conflicts.

### Supplemental Material

Tables S1–S4

Figure S1

STROBE Checklist
